# Integrated network analysis of transcriptomic and proteomic data in psoriasis

**DOI:** 10.1186/1752-0509-4-41

**Published:** 2010-04-08

**Authors:** Eleonora Piruzian, Sergey Bruskin, Alex Ishkin, Rustam Abdeev, Sergey Moshkovskii, Stanislav Melnik, Yuri Nikolsky, Tatiana Nikolskaya

**Affiliations:** 1 Vavilov Institute of General Genetics, Russian Academy of Sciences, Gubkina St, 3 GSP-1, 119991 Moscow, Russia; 2 GeneGo, Inc, Saint Joseph, MI, USA; 3 Center for Theoretical Problems of Physico-Chemical Pharmacology, Russian Academy of Sciences, Kosigina str, 4, 119991 Moscow, Russia; 4 Institute of Biomedical Chemistry, Russian Academy of Medical Sciences, 10 Pogodinskaya st, 119121 Moscow, Russia

## Abstract

**Background:**

Psoriasis is complex inflammatory skin pathology of autoimmune origin. Several cell types are perturbed in this pathology, and underlying signaling events are complex and still poorly understood.

**Results:**

In order to gain insight into molecular machinery underlying the disease, we conducted a comprehensive meta-analysis of proteomics and transcriptomics of psoriatic lesions from independent studies. Network-based analysis revealed similarities in regulation at both proteomics and transcriptomics level. We identified a group of transcription factors responsible for overexpression of psoriasis genes and a number of previously unknown signaling pathways that may play a role in this process. We also evaluated functional synergy between transcriptomics and proteomics results.

**Conclusions:**

We developed network-based methodology for integrative analysis of high throughput data sets of different types. Investigation of proteomics and transcriptomics data sets on psoriasis revealed versatility in regulatory machinery underlying pathology and showed complementarities between two levels of cellular organization.

## Background

*Psoriasis vulgaris *is one of the most prevalent chronic inflammatory skin diseases affecting approximately 2% of individuals in Western societies, and found worldwide in all populations. Psoriasis is a complex disease affecting cellular, gene and protein levels and presented as skin lesions. The skin lesions are characterized by abnormal keratinocyte differentiation, hyperproliferation of keratinocytes, and infiltration of inflammatory cells [1,2]. The mechanisms of psoriasis pathology are complex and involve genetic and environmental factors. As we gain more knowledge about molecular pathways implicated in the disease, novel therapies emerge (such as etanercept and infliximab that target TNF-α or CD11a- mediated pathways [3,4]). In recent years, microarray mRNA expression profiling [5-8] of lesional psoriatic skin revealed over 1,300 differentially expressed genes. Enrichment analysis (EA) showed that these genes encode proteins involved in regeneration, hyperkeratosis, metabolic function, immune response, and inflammation and revealed a number of modulating signaling pathways. These efforts may help to develop new-generation drugs.

However, enrichment analysis limits our understanding of altered molecular interactions in psoriasis as it provides a relative ranking based on ontology terms resulting in the representation of fragmented and disconnected perturbed pathways. Furthermore, analysis of gene expression alone is not sufficient for understanding the whole variety of pathological changes at different levels of cellular organization. Indeed, new methodologies have been applied to the analysis of OMICs data in complex diseases that include algorithm-based biological network analysis [9-13] and meta-analysis of multiple datasets of different types [14-19]. Here, we applied several techniques of network and meta-analysis to reveal the similarities and differences between transcriptomics- and proteomics-level perturbations in psoriasis lesions. We particularly focused on revealing novel regulatory pathways playing a role in psoriasis development and progression.

## Methods

### Skin biopsies

Acquisition of the human tissue was approved by the Vavilov Institute of General Genetics of Russian Academy of Sciences review board and the study was conducted after patient's consent and according to the Declaration of Helsinki Principles.

A total of 6 paired nonlesional and lesional (all were plaque-type) skin biopsies from 3 psoriatic patients were profiled using 2D electrophoresis. All the donors who gave biopsy tissue (both healthy controls and individuals with psoriasis) provided a written informed consent for the tissue to be taken and used in this study. Clinical data for all patients are listed in Additional file 1.

Full-thickness punch biopsies were taken from uninvolved skin (at least 2 cm distant from any psoriatic lesion; 6 mm diameter) and from the involved margin of a psoriatic plaque (6 mm diameter) from every patient.

### Sample preparation

Skin biopsies from lesional (n = 3) and non-lesional (n = 3) skin (~50 mg each) were mechanically homogenized on ice with mortar and pestle in solubilization buffer comprised of 7 M urea (Fisher Scientific), 2 M thiourea (MERK), 4% (w/v) CHAPS (Fluka), 0.5% (w/v) Triton X-100 (ICN), 0.5%(w/v) ampholines 3/10 (Bio-Rad), 20 mM Tris base (Fisher Scientific), 1 mM MgCl_2 _(Fluka), and 0.2 mM PMSF (Acros). The amount of solubilization buffer taken per sample was seven times tissue wet weight. Samples were then carefully sonicated at 4°C for 15 sec (3 bursts for 5 sec) with Bandelin sonicator at 50% power output. Following homogenization and sonication DTT (Acros) was added to reach the final concentration of 65 mM, and samples left at 4°C for 30 min. Then Na_2_EDTA was added (2 mM final) and mixture was incubated for additional 2 h and then centrifuged at *12,000 × g *for 10 min to remove insoluble debris. Relative protein concentration was determined with GS-800 Calibrated Densitometer (Bio-Rad) after 1D-SDS-PAGE of 3 μl sample solution loaded on 8 × 9 cm mini-gel stained with Coomassie R-250 after electrophoresis.

### Two-dimensional electrophoresis

For the first dimension CA-IEF method was used [20]. Glass tubes 20 × 1.5 mm (Bio-Rad) was filled with gel mixture containing 8.3 M urea (Fisher Scientific), 4% (w/v) acrylamide monomers (Acros), 2% CHAPS (Fluka), 1.6% (w/v) 5/8 and 0.4% (w/v) 3/10 ampholines (Bio-Rad), loaded with 120 μg protein and run 15 min at 200 V, 30 min at 300 V, 16 hrs at 400 V, and 1 h at 800 V. Isoelectrofocusing was performed in Protean II xi cell (Bio-Rad). Before second dimension gels were extruded from tubes and equilibrated in 60 mM Tris-buffer pH 6.8, containing 2% (w/v) SDS, 10% (w/v) glycerol, and 1% (w/v) DTT for 30 min. For protein separation in the second dimension, 9-16% linear gradient slab gels prepared according to the standard protocol [20] were used.

### Gel image analysis

Protein spots on 2-DE gels were visualized by silver staining [21] and scanned with resolution 300 dpi. Images were analyzed using Melanie III software (GeneBio, Switzerland). The conventional analysis involved (i) protein spot relative volume (%Vol) determination, which was expressed as the sum of pixel intensities in the certain spot divided to the sum of pixel intensities in all spots on the gel; (ii) gel alignment; and (iii) spot matching. Further, sets of %Vol values for every spot were processed by Student test, thereby testing whether there was a significant variation of the certain protein level between two specified groups.

### Protein identification by MALDI-TOF mass-spectrometry

Protein spots were cut out (~3 mm3) from 2-DE gels, destained, and in-gel digested with trypsin. Mass-spectrometry of trypsin digested proteins (spots No. 1,2,3,4) was performed using a Microflex MALDI-TOF mass-spectrometer (Bruker, Germany). Peptide samples (0.2-1 μl) were mixed with an equal volume of 2,5-dihydroxybenzoic acid solution (20 mg/ml; Sigma, USA) in 20% acetonitrile and 0.1% trifluoroacetic acid, and the resulting droplets were dried in air. Mass-spectra were obtained for mass range from 800 to 4000 daltons in reflection mode and calibrated using internal standards (trypsin autolysis peaks, MH+ 1046.54, 2212.10 daltons). Peptide peak lists were formed by the SNAP algorithm (XMass software, Bruker). Proteins were identified using the Mascot database search engine. The search parameters were as follows: mass tolerance 100 ppm, NCBI protein sequence database, *Homo sapiens *taxon, one missed cleavage, variable modifications by propionamide for cysteines and oxidation for methionines.

Low molecular weight proteins (9-20 kDa, spots No. 5,6,7,8,9,10) were identified using nanoLC-MS/MS mass-spectrometry for higher convenience, regarding lack of cleavage sites in such proteins. Analysis of trypsin digest was performed on electrospray ion trap (XCT Ultra Ion Trap Chip Cube 6330 series, Agilent) equipped with chip-cube head. One μl of each sample was subjected (flow rate 3 μl/min) onto reverse-phase in-chip column (40 nl capacity) for 10 minutes under isocratic buffer (5% acetonitrile in 0.1% TFA). Following sample application peptides were separated by linear gradient (0.3 μl/min) of solution A (0.1% TFA/water) and solution B (80% ACN/0.1% TFA/water) for 60 minutes. Spectrum scanning was repeated three times for each sample of protein gel spots and tissue hydrolysate. Mass spectra of eluted peptide were simultaneously obtained under positive polarity for 425-1325 m/z range both in MS and MS/MS mode, 2.1 kV applied accumulation of 85000 ions for 50 milliseconds, averages on 2 spectra. Mass spectra were processed with Spectrum Mill MS Proteomics workbench software (Agilent). Proteins were identified using SwissProt Human Database in the following parameters: score ≥ 7 for peptide and ≥ 20 for protein, minimum S/N ratio 20, maximum peptide ion charge +4, precursor mass tolerance ± 2.5 Da, product mass tolerance ± 0.7 Da, Proteins identification was accomplished with detection of minimum 3 peaks of the same peptide ion with maximum of 2 missed cleavages.

### Microarray data analysis

We used recently published data set [22] from GEO data base (http://www.ncbi.nlm.nih.gov/geo/; accession number GSE14095). We compared 28 pairs of samples (in each pair there was a sample of lesional skin and a sample of healthy skin from the same patient). Values for each sample were normalized by sample median value in order to unify distributions of expression signals. For assessment of differential expression we used paired Welch t-test with FDR correction [23]. Probe set was considered as differentially expressed if its average fold change exceeded 2.5 and FDR corrected p-value was less than 0.01.

### Overconnection analysis

All network-based analyses were conducted with MetaCore software suite http://www.genego.com. This software employs a dense and manually curated database of interactions between biological objects and variety of tools for functional analysis of high-throughput data.

We defined a gene as overconnected with the gene set of interest if the corresponding node had more direct interactions with the nodes of interest than it would be expected by chance. Significance of overconnection was estimated using hypergeometric distribution with parameters r - number of interactions between examined node and the list of interest; R - degree of examined node, n -sum of interactions involving genes of interest and N - total number of interactions in the database:

### Hidden nodes analysis

In addition to direct interacting objects, we also used objects that may not interact directly with objects of interest but are important upstream regulators of those[24]. The approach is generally the same as described above, but the shortest paths instead of direct links are taken into account. As we were interested in transcriptional regulation, we defined a transcriptional activation shortest path as the preferred shortest path from any object in the MetaCore database to the transcription factor target object from the data set. We added an additional condition to include the uneven number of inhibiting interactions in the path (that's required for the path to have activating effect). If the number of such paths containing examined gene and leading to one of objects of interest were higher than expected by chance, this gene was considered as significant hidden regulator. The significance of a node's importance was estimated using hypergeometric distribution with parameters r - number of shortest paths between containing currently examined gene; R - total number of shortest paths leading to a gene of interest through transcriptional factor, n -total number of transcription activation shortest paths containing examined gene and N - total number of transcription activation shortest paths in the database.

### Rank aggregation

Both topology significance approaches produced lists of genes significantly linked to a gene or protein set of interest, ranked by corresponding p-values. To combine results of these two approaches, we used a weighted rank aggregation method described in [25]. Weighted Spearman distance was used as distance measure and the genetic algorithm was employed to select the optimal aggregated list of size 20. This part of work was accomplished in R 2.8.1 http://www.r-project.org.

### Network analysis

In addition to topology analysis, we examined overexpressed genes and proteins using various algorithms for selecting connected biologically meaningful subnetworks enriched with objects of interest. Significance of enrichment is estimated using hypergeometric distribution.

We first used an algorithm intended to find regulatory pathways that are presumably activated under pathological conditions. It defines a set of transcription factors that are directly regulating genes of interest and a set of receptors whose ligands are in the list of interest and then constructs series of networks; one for each receptor. Each network contains all shortest paths from a receptor to the selected transcriptional factors and their targets. This approach allows us to reveal the most important areas of regulatory machinery affected under the investigated pathological condition. Networks are sorted by enrichment p-value.

The second applied algorithm used was aimed to define the most influential transcription factors. It considers a transcriptional factor from the data base and gradually expands the subnetwork around it until it reaches a predefined threshold size (we used networks of 50 nodes). Networks are sorted by enrichment p-value.

## Results

### Differentially abundant proteins

**Figure 1:**
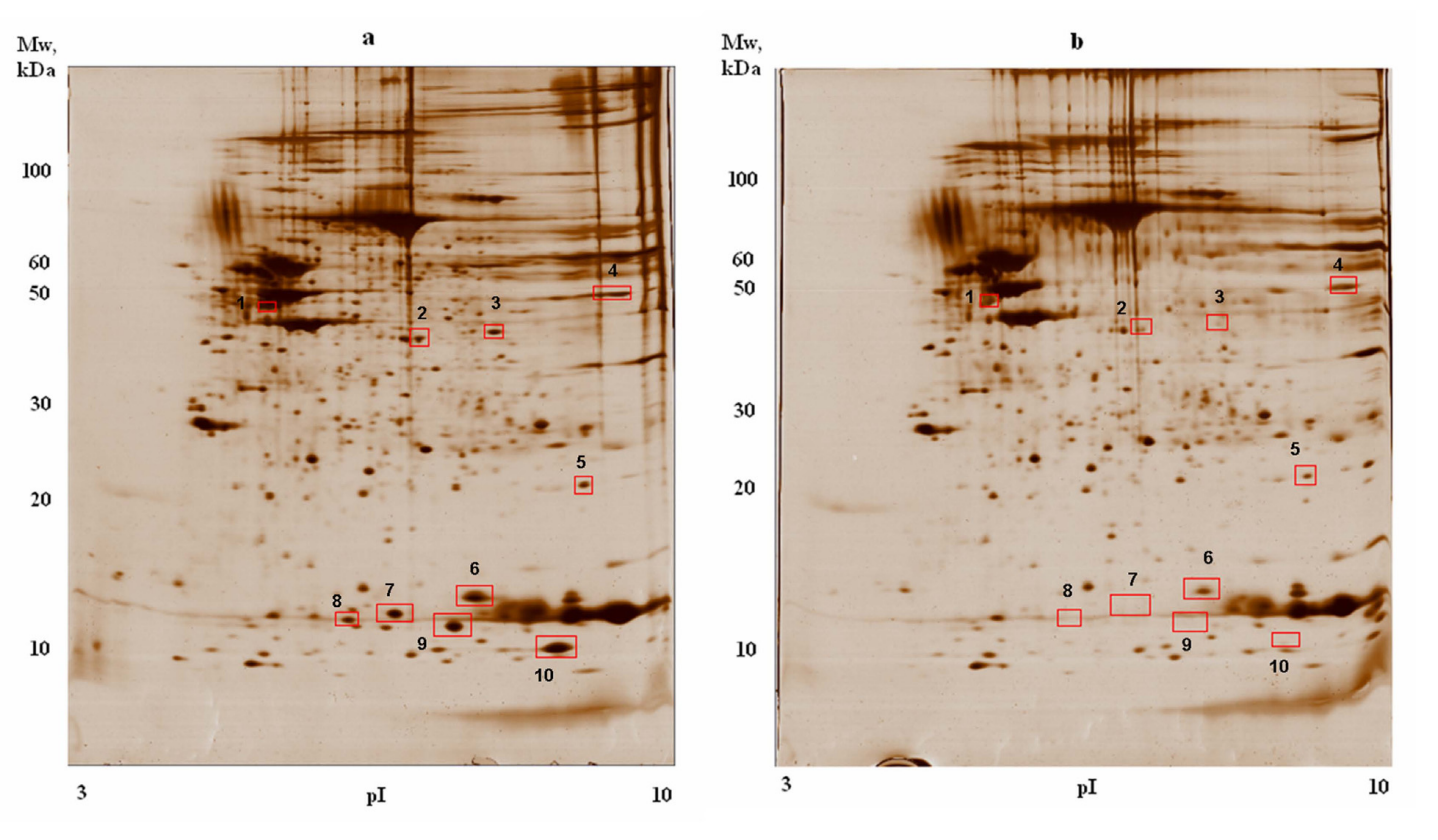
**Representative silver-stained 2DE gel images of lesional and uninvolved skin biopsy lysates**. a) - gel image of lesional skin biopsy lysate; b) - gel image of uninvolved skin biopsy lysate. Spots corresponding to proteins overexpressed in lesions are marked with red rectangles and numbered. Spot 1 correspond to 3 proteins of keratin family, spot 2 - SCCA2, spot 3 - SCCA1, spot 4 - enolase 1, spot 5 - SOD2, spot 6 - galectin-7. S100A7 is found in spots 7 and 8 and S100A9 corresponds to 9^th ^and 10^th ^spots.

Protein abundance was determined by densitometric quantification of the protein spots on 2D-electophoresis gel (Figure 1; see also Additional file 4) followed by MALDI-TOF mass spectrometry. Total of 10 proteins were over-abundant at least 2-fold in lesional skin compared with uninvolved skin: Keratin 14, Keratin 16, Keratin 17, Squamous cell carcinoma antigen, Squamous cell carcinoma antigen-2, Enolase 1, Superoxide dismutase [Mn], Galectin-7, S100 calcium-binding protein A9 and S100 calcium-binding protein A7 (Table 1). Several of these proteins were previously reported to be over-abundant in psoriatic plaques [26-29].

**Table 1:** Proteins with elevated expression in lesions

Protein name	Gene	Fold change
Keratin 17	KRT17	10.94444444
Keratin 14	KRT14	10.94444444
Keratin 16	KRT16	10.94444444
SCCA2/SCCA1 fusion protein isoform 2	SERPINB4	4.242424242
squamous cell carcinoma antigen; SCC antigen	SERPINB3	11.66666667
Enolase 1	ENO1	2.175
Superoxide dismutase [Mn]	SOD2	2
Galectin-7 (Gal-7) (HKL-14) (PI7) (p53-induced protein 1)	LGALS7B	6
Protein S100-A9 (S100 calcium-binding protein A9)	S100A9	Lesion only
Protein S100-A7 (S100 calcium-binding protein A7) (Psoriasin)	S100A7	Lesion only

The proteins belonged to a diverse set of pathways and processes. Thus, keratin 17, keratin 14, and keratin 16 are a member of the keratin gene family. The keratins are intermediate filament proteins responsible for the structural integrity of epithelial cells. SERPINB4 and SERPINB3 are serine protease inhibitor to modulate the host immune response against tumor cells. Enolase 1, more commonly known as alpha-enolase, is a glycolytic enzyme expressed in most tissues, one of the isozymes of enolase. Superoxide dismutase 2 protein (SOD2) transforms toxic superoxide, a byproduct of the mitochondrial electron transport chain, into hydrogen peroxide and diatomic oxygen. Galectins are a family of beta-galactoside-binding proteins implicated in modulating cell-cell and cell-matrix interactions. Differential and in situ hybridizations indicate that this lectin is specifically expressed in keratinocytes. The cellular localization and its striking down-regulation in cultured keratinocytes imply a role in cell-cell and/or cell-matrix interactions necessary for normal growth control. S100A9 and S100A7 proteins are localized in the cytoplasm and/or nucleus of a wide range of cells, and involved in the regulation of a number of cellular processes such as cell cycle progression and differentiation. S100A7 is markedly over-expressed in the skin lesions of psoriatic patients.

**Figure 2:**
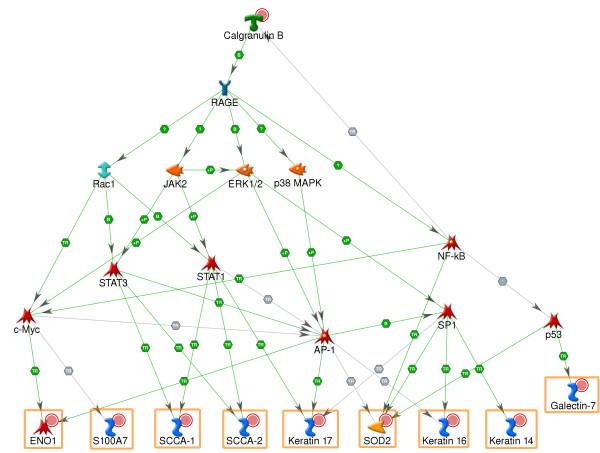
**Network illustrating regulatory pathways leading to transcription activation of proteomics markers**. Red circles denote upregulated proteins. Designations of network objects and interaction types can be found in Additional file 4.

We attempted to connect the proteins into a network using a collection of over 300,000 manually curated protein interactions and several variants of "shortest path" algorithms applied in MetaCore suite [30] (Figure 2, see Methods for details). The genes encoding overabundant proteins were found to be regulated by several common transcription factors (TFs) including members of the NF-kB and AP-1 complexes, STAT1, STAT3, c-Myc and SP1. Moreover, the upstream pathways activating these TFs were initiated by the overabundant S100A9 through its receptor RAGE [31] and signal transduction kinases (JAK2, ERK, p38 MAPK). This network also included a positive feedback loop as S100A9 expression was determined to be controlled by NF-kB[32]. The topology of this proteomics-derived network was confirmed by several transcriptomics studies [33-38] which showed overexpression of these TFs in psoriasis lesions. Transiently expressed TFs normally have low protein level and, therefore, usually fail to be detected by proteomics methods.

RAGE receptor is clearly the key regulator on this network and plays the major role in orchestrating observed changes of protein abundance. This protein is abundant in both keratinocytes and leukocytes, though normally its expression is low [39]. RAGE participates in a range of processes in these cell types, including inflammation. It is being investigated as a drug target for treatment of various inflammatory disorders [40]. Thus, we may propose that RAGE can also play significant role in psoriasis.

### Differentially expressed genes

We used Affymetrix gene expression data set from the recent study [22] involving 33 psoriasis patients. Originally, more than 1300 probe sets were found to be upregulated in lesions as compared with unlesional skin of the same people. We identified 451 genes overexpressed in lesional skin under more stringent statistical criteria (28 samples of lesional skin were matched with their non-lesional counterparts from the same patients in order to exclude individual expression variations, genes with fold change >2.5 and FDR-adjusted p-value < 0.01 were considered as upregulated). The list of overexpressed genes can be found in Additional file 2. The genes encoding 7 out of 10 proteomic markers were overexpressed, well consistent with proteomics data. Expression of Enolase 1, Keratin 14 and Galectin 7 was not altered.

### Common transcription regulation for overexpressed genes and differentially abundant proteins

**Figure 3:**
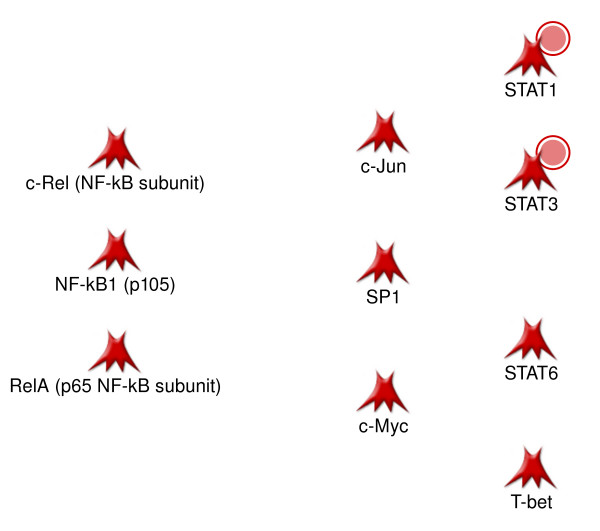
**Common transcriptional factors important for regulation of objects at both transcriptomics and proteomic levels**. Objects in MetaCore database representing transcriptional factors found to be important regulators of pathology-related genes. Red circles denote that corresponding gene is upregulated in psoriatic lesion. Designations of network objects and interaction types can be found in Additional file 4.

Despite good consistency between the proteomics and expression datasets, the two orders of magnitude difference in list size make direct correlation analysis difficult. Therefore, we applied interactome methods for the analysis of common upstream regulation of the two datasets at the level of transcription factors. First, we defined the sets of the most influential transcription factors using two recently developed methods of interactome analysis [41] and the "hidden nodes" algorithm [42]. The former method ranks TFs based on their one-step overconnectivity with the dataset of interest compared to randomly expected number of interactions. The latter approach takes into account direct *and *more distant regulation, calculating the p-values for local subnetworks by an aggregation algorithm [42]. We calculated and ranked the top 20 TFs for each data type and added several TFs identified by network analysis approaches (Table 2). The TFs common for both data types were taken as set of 'important pathological signal transducers' (Figure 3). Noticeably, they closely resemble the set of TFs regulating the protein network on Figure 2.

**Table 2:** Common transcriptional factors for proteomics and expression datasets

Transcriptomics				Proteomics			
Entrez ID	Gene	Over-connection P-Value	Hidden nodes P-Value	Entrez ID	Gene	Over-connection P-Value	Hidden nodes P-Value
**6772**	**STAT1**	**4.081E-28**	**1.027E-22**	2736	GLI2	4.318E-03	2.080E-04
3659	IRF1	1.344E-22	3.203E-19	**6772**	**STAT1**	**2.234E-04**	**1.293E-04**
**4790**	**NFKB1**	**3.677E-13**	**2.523E-25**	3091	HIF1A	2.345E-03	2.402E-04
**5970**	**RELA**	**3.726E-14**	**4.120E-24**	6778	STAT6	9.420E-04	2.080E-04
**5966**	**REL**	**4.844E-17**	**3.580E-16**	7392	USF2	1.507E-04	-
6688	SPI1	7.019E-13	1.245E-16	**6774**	**STAT3**	**2.542E-05**	**-**
3394	IRF8	7.594E-12	1.903E-16	6776	STAT5A	-	2.820E-05
1051	CEBPB	1.590E-12	1.271E-15	668	FOXL2	1.100E-02	1.388E-03
2908	NR3C1	2.307E-05	1.504E-17	2078	ERG	2.386E-04	1.388E-03
10379	IRF9	8.193E-05	4.037E-17	**6777**	**STAT5B**	**-**	**2.820E-05**
3660	IRF2	3.794E-12	3.914E-09	7020	TFAP2A	4.806E-04	-
6434	SFRS10	-	4.420E-17	5371	PML	1.177E-03	-
6775	STAT4	1.637E-03	5.842E-16	2735	GLI1	1.053E-02	-
**6667**	**SP1**	**5.508E-12**	**2.013E-07**	**6667**	**SP1**	**3.269E-05**	**-**
**6777**	**STAT5B**	**2.725E-04**	**2.063E-15**	**3725**	**JUN**	**1.198E-03**	**-**
**3725**	**JUN**	**2.182E-11**	**7.521E-11**	8462	KLF11	6.710E-03	2.775E-03
**6774**	**STAT3**	**9.643E-10**	**2.114E-11**	2099	ESR1	1.650E-02	-
7157	TP53	9.352E-10	7.171E-08	10538	BATF	1.765E-02	-
**30009**	**TBX21**	**5.119E-03**	**1.594E-14**	**30009**	**TBX21**	**-**	**1.911E-04**
1874	E2F4	1.537E-09	8.737E-07	7421	VDR	-	1.693E-04
**Additional TFs identified by subnetwork-based analysis**							
**4609**	**MYC**	**2.680E-69**	**-**	**4609**	**MYC**	**1.120E-07**	**-**
				**5966**	**REL**	**1.120E-07**	**-**
				**5970**	**RELA**	**1.120E-07**	**-**
				**4790**	**NFKB1**	**1.120E-07**	**-**

Some genes were considered significant only by one of two topological approaches (this is evident for proteomics data, where low number of proteins limits capabilities of topological analysis). Missing p-value means that correspondent gene has not passed 0.05 significance threshold and has been listed among top factors only due to low p-value for other topological approach. Only one p-value was determined in the case of network analysis (enrichment p-value for the network built around seed transcription factor). Transcriptional factors common for proteomics and transcriptomics level are in bold text.

### Identification of influential receptors

In the next step, we applied "hidden nodes" algorithm to identify the most influential receptors that could trigger maximal possible transcriptional response. In total, we found 226 membrane receptors significantly involved into regulation of 462 differentially expressed genes ('hidden nodes' p-value < 0.05). The complete list of receptors can be found in Additional file 3. Assuming that topological significance alone does not necessarily prove that all receptors are involved in real signaling or are even expressed in the sample; we filtered this list by expression performance. The receptors used were those whose encoding genes or corresponding ligands were overexpressed greater than 2.5 fold. We assumed that the pathways initiated by over-expressed receptors and ligands are more likely to be activated in psoriasis. Here we assumed that expression alterations and protein abundance are at least collinear. An additional criterion was that the candidate receptors had to participate in the same signaling pathways with at least one of the common TFs. No receptor was rejected based on this criterion.

**Figure 4:**
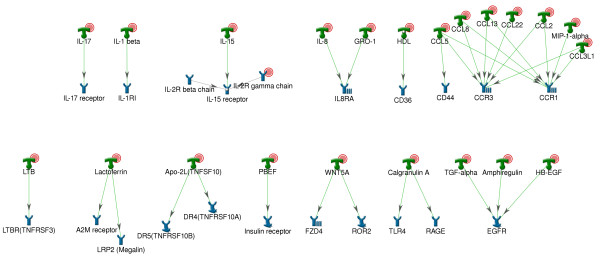
**Candidate receptors with their respective upregulated ligands**. Initial steps of pathways presumably activated in lesions (ligands, overexpressed at transcriptional level and their corresponding receptors) Red circles denote that corresponding gene is upregulated in psoriatic lesion. Designations of network objects and interaction types can be found in Additional file 4.

**Figure 5:**
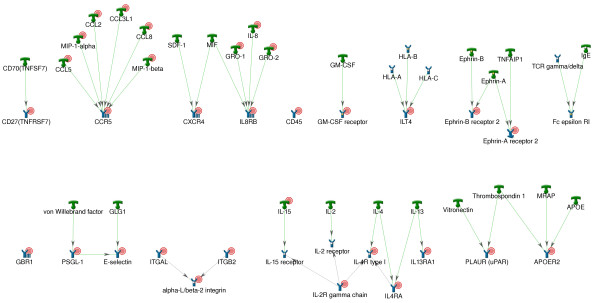
**Upregulated candidate receptors with their respective ligands**. Initial steps of pathways presumably activated in lesions (receptors, overexpressed at transcriptional level and their corresponding ligands) Red circles denote that corresponding gene is upregulated in psoriatic lesion. Designations of network objects and interaction types can be found in Additional file 4.

In total, 44 receptors passed the transcription cut-off. Of these 24 receptor genes were overexpressed; 23 had overexpressed ligands and 3 cases had overexpression of both ligands and receptors (IL2RB, IL8RA and CCR5; see Figures 4 and 5 and Additional file 4). Interestingly, for several receptors, more than one ligand was over-expressed (Figure 4). Several receptors are composed of several subunits, only one of which was upregulated (for example, IL-2 receptor has only gamma subunit gene significantly upregulated).

Out of 44 receptors we identified by topology analysis, 21 were previously reported as psoriasis markers (they are listed in Table 3 with corresponding references). The other 23 receptors were not reported to be linked to psoriasis or known to be implicated in other inflammatory diseases. These receptors belong to different cellular processes (development, cell adhesion, chemotaxis, apoptosis and immune response) (Table 3).

**Table 3:** Receptors identified in our study and not yet studied in connection to psoriasis

Gene	Protein	Entrez Gene ID	Connection to psoriasis
EPHA2	Ephrin-A receptor 2	1969	No
EPHB2	Ephrin-B receptor 2	2048	No
FCER1G	Fc epsilon RI gamma	2207	No
INSR	Insulin receptor	3643	No
LTBR	LTBR(TNFRSF3)	4055	No
PLAUR	PLAUR (uPAR)	5329	No
TNFRSF10A	DR4(TNFRSF10A)	8797	No
TNFRSF10B	DR4(TNFRSF10A)	8795	No
CD44	CD44	960	Possible[55]
CSF2RB	CSF2RB	1439	Possible[56]
CXCR4	CXCR4	7852	Possible[57]
FZD4	FZD4	8322	Possible[58]
GABBR1	GBR1	2550	Possible[59]
IL10RA	IL10RA	3587	Possible[60]
IL13RA1	IL13RA1	3597	Possible[61]
IL2RB	IL-2R beta chain	3560	Possible[62]
IL2RG	IL-2R gamma chain	3561	Possible[62]
IL4R	IL4RA	3566	Possible[63]
LILRB2	ILT4	10288	Possible[64]
LRP2	LRP2 (Megalin)	4036	Possible[65]
LRP8	APOER2	7804	Possible[65]
ROR2	ROR2	4920	Possible[58]
AGER	RAGE	177	Yes[66]
CCR1	CCR1	1230	Yes[67]
CCR2	CCR2	1231	Yes[68]
CCR3	CCR3	1232	Yes[69]
CCR5	CCR5	1234	Yes[70]
CD2	CD2	914	Yes[71]
CD27	CD27(TNFRSF7)	939	Yes[72]
CD36	CD36	948	Yes[73]
CD3D	CD3 delta	915	Yes[74]
EGFR	EGFR	1956	Yes[75]
IL17RA	IL-17 receptor	23765	Yes[76]
IL1R1	IL-1RI	3554	Yes[77]
IL8RA	IL8RA	3577	Yes[78]
IL8RB	IL8RB	3579	Yes[78]
ITGAL	ITGAL	3683	Yes[79]
ITGB2	ITGB2	3689	Yes[80]
LRP1	A2 M receptor	4035	Yes[81]
PTPRC	CD45	5788	Yes[82]
SDC3	Syndecan-3	9672	Yes[83]
SELE	E-selectin	6401	Yes[84]
SELPLG	PSGL-1	6404	Yes[85]
TLR4	TLR4	7099	Yes[86]

'Possible' term was used if protein name co-occurred with psoriasis in articles, but no clear evidence of its implication was shown. In some cases, ligands are associated with psoriasis (i.e, IL-10).

## Discussion

Meta-analysis of multiple OMICs data types and studies is becoming an important research tool in understanding complex diseases. Several methods were developed for correlation analysis between the datasets of different type, such as mRNA and proteomics [18,43-46]. However, there are many technological challenges to resolve, including mismatching protein IDs and mRNA probes, fundamental differences in OMICs technologies, differences in experimental set-ups in studies done by different groups etc [47]. Moreover, biological reasons such as differences in RNA and protein degradation processes also contribute to variability of different data types. As a result, transcriptome and proteome datasets usually show only weak positive correlation although were considered as complimentary. More recent studies focused on functional similarities and differences observed for different levels of cellular organization and reflected in different types of OMICs data [48-51]. For example, common interacting objects were found for distinct altered transcripts and proteins in type 2 diabetes [52]. In one leukemia study [53] authors found that distinct alterations at transcriptomics and proteomic levels reflect different sides of the same deregulated cellular processes.

The overall concordance between mRNA and protein expression landscapes was addressed in earlier studies, although the data types were compared mostly at the gene/protein level with limited functional analysis [14,47]. Later, ontology enrichment co-examination of transcriptomics and proteomic data has shown that the two data types affect similar biological processes and are complimentary [49,53,54]. However, the key issue of biological causality and functional consequences of distinct regulation events at both mRNA and protein levels of cellular organization were not yet specifically addressed. These issues cannot be resolved by low resolution functional methods like enrichment analysis. Instead, one has to apply more precise computational methods such as topology and biological networks, which take into consideration directed binary interactions and multi-step pathways connecting objects between the datasets of different types regardless of their direct overlap at gene/protein level [12,13]. For example, topology methods such as "hidden nodes" [24,41] can identify and rank the upstream regulatory genes responsible for expression and protein level alterations while network tools help to uncover functional modules most affected in the datasets, identify the most influential genes/proteins within the modules and suggest how specific modules contribution to clinical phenotype [10,52].

In this study, we observed substantial direct overlap between transcriptomics and proteomics data, as 7 out of 10 over-abundant proteins in psoriasis lesions were encoded by differentially over-expressed genes. However, the two orders of magnitude difference in dataset size (462 genes versus 10 proteins) made the standard correlation methods inapplicable. Besides, proteomics datasets display a systematic bias in function of abundant proteins, favoring "effector" proteins such as structural, inflammatory, core metabolism proteins but not the transiently expressed and fast degradable signaling proteins. Therefore, we applied topological network methods to identify common regulators for two datasets such as the most influential transcription factors and receptors. We have identified some key regulators of the "proteomics" set among differentially expressed genes, including transcription factors, membrane receptors and extracellular ligands, thus reconstructing upstream signaling pathways in psoriasis. In particular, we identified 24 receptors previously not linked to psoriasis.

Importantly, many ligands and receptors defined as putative starts of signaling pathways were activated by transcription factors at the same pathways, clearly indicating on positive regulatory loops activated in psoriasis. The versatility and the variety of signaling pathways activated in psoriasis is also impressive, which is evident from differentially overexpression of 44 membrane receptors and ligands in skin lesions. This complexity and redundancy of psoriasis signaling likely contributes to the inefficiency of current treatments, even novel therapies such as monoclonal antibodies against TNF-α and IL-23. Thus, the key regulator, RAGE receptor, triggers multiple signaling pathways which stay activated even when certain immunological pathways are blocked. Our study suggests that combination therapy targeting multiple pathways may be more efficient for psoriasis (particularly considering feasibility for topical formulations). In addition, the 24 receptors we identified by topology analysis and previously not linked with psoriasis can be tested as potential novel targets for disease therapy.

The functional machinery of psoriasis is still not complete and additional studies can be helpful in "filling the gaps" of our understanding of its molecular mechanisms. For instance, kinase activity is still unaccounted for, as signaling kinases are activated only transiently and are often missed in gene expression studies. Topological analysis methods such as "hidden nodes" [24] may help to reconstruct regulatory events missing in the data. Also, the emerging phosphoproteomics methodology may prove to become a helpful and complimentary OMICs technology. The network analysis methodology is not dependent on the type of data analyzed and or any gene/protein content overlap between the studies and is well applicable for functional integration of multiple data types.

## Conclusion

We have successfully applied network-based methods to integrate and explore two distinct high-throughput disease data sets of different origin and size. Through identification of common regulatory machinery that is likely to cause overexpression of genes and proteins, we came to the signaling pathways that might contribute to the altered state of regulatory network in psoriatic lesion. Our approach allows easy integrative investigation of different data types and produces biologically meaningful results, leading to new potential therapy targets. We have demonstrated that pathology can be caused and maintained by a great amount of various cascades, many previously not described as implicated in psoriasis; therefore, combined therapies targeting multiple pathways might be effective in treatment.

## Authors' contributions

EP and TN conceived the study and participated in its design and coordination; SB, SM and StM performed the proteomics analysis of psoriatic tissues, AI performed statistical and network-based analyses and wrote the manuscript, RA helped with bioinformatical analysis of the data, YN edited the manuscript. All authors have read and approved the manuscript.

## Supplementary Material

Additional file 1 **Table S1**. Patient description Click here for file

Additional file 2 **Table S2**. List of genes significantly upregulated in lesions at the mRNA level Click here for file

Additional file 3 **Table S3**. List of receptors significantly involved in regulation of genes overexpressed in psoriatic plaque ('hidden nodes' algorithm result) Click here for file

Additional file 4 **Legend**. Network layout legend explaining meaning of object icons and interaction types Click here for file
