# Supplementary material for: Integrated network analysis of transcriptomic and proteomic data in psoriasis
Source: BMC Syst Biol. 2010 Apr 8;4:41. doi: 10.1186/1752-0509-4-41 (PMC2873316; doi:10.1186/1752-0509-4-41)
Supplement: Additional file 1 — Table S1. Patient description [file 1752-0509-4-41-S1.DOCX]

Table S1. Patients

| Patient | Sex | Sample | Age (years) | Duration (years) | Family history | PASI | PsA |
| --- | --- | --- | --- | --- | --- | --- | --- |
| 1 | F | L/NL | 28 | >10 | Y | 6.8 | No |
| 2 | M | L/NL | 39 | >10 | N | 7.2 | No |
| 3 | F | L/NL | 30 | >10 | N | 6.9 | No |

L - lessional; NL – nonlessional; M – male; F – female; PsA – psoriatic arthritis
