# Supplementary material for: Integrated network analysis of transcriptomic and proteomic data in psoriasis
Source: BMC Syst Biol. 2010 Apr 8;4:41. doi: 10.1186/1752-0509-4-41 (PMC2873316; doi:10.1186/1752-0509-4-41)
Supplement: Additional file 4 — Legend. Network layout legend explaining meaning of object icons and interaction types [file 1752-0509-4-41-S4.PDF]

Network Objects

Enzymes			
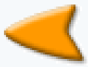	Generic enzyme		
**KINASE**		**PHOSPHATASE**	
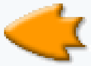	Generic kinase	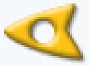	Generic phosphatase
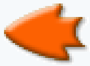	Protein kinase	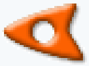	Protein phosphatase
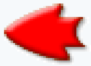	Lipid kinase	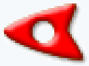	Lipid phosphatase
**PHOSPHOLIPASE**			
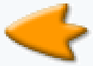	Generic phospholipase		
**PROTEASE**		**GTPase**	
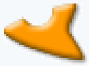	Generic protease	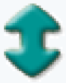	G-alpha
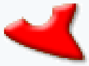	Metalloprotease	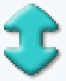	RAS - superfamily

Generic classes	
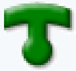	Receptor ligand
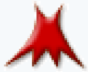	Transcription factor
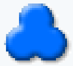	Protein
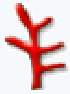	Cell membrane glycoprotein
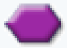	Compound
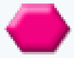	Predicted metabolite or user’s structure
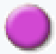	Inorganic ion
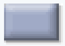	Reaction
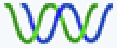	DNA
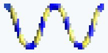	RNA
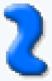	Generic binding protein

	Channels / Transporters
	Generic channel
	Ligand-gated ion channel
	Voltage-gated ion channel
	Transporter

	Receptors
	Generic receptor
	GPCR
	Receptors with enzyme activity

	G protein adaptor / regulators
	G beta/gamma
	Regulators (GDI, GAP, GEF)

	Groups of objects
	**A complex or a group**<br>Proteins or compounds physically connected into a complex or related as a group
	**Logical association**<br>Related proteins or compounds are connected into groups. To see the relations (logical associations) within a group, use «Expand group» function in the scroll-down right-button menu. Use «Collapse logical relations» function to close the group.

	Expression data
	**Overexpressed gene(s)**<br>Genes with higher conditional expression level compared to the experimental "control"
	**Underexpressed gene(s)**<br>Genes with lower conditional expression level compare to the experimental "control"

Interactions between objects

Link legend	
	Positive effect
	Negative effect
	Unspecified effect
	Technical link

Mechanisms	
**Physical interactions**	
B	Binding<br>Protein or compound binds other protein or compound
C	Cleavage<br>Cleavage of a protein at a specific site yielding distinctive peptide fragments.<br>Proteolytic cleavage can be carried out by both enzymes and compounds
CM	Covalent modifications<br>(neddylation/deneddylation, sumoylation/desumoylation, ubiquitination/deubiquitination and etc.) Protein activity regulation by covalent binding of a small chemical group to the aminoacids of an active site.
+P	Phosphorylation<br>Protein activity regulation by an addition of a phosphate group
-P	Dephosphorylation<br>Protein activity regulation by a removal of a phosphate group
T	Transformation<br>Protein activity regulation by binding & hydrolysis of GTP
Tn	Transport<br>Transport of a protein or a compound between organelles

Z	**Catalysis**<br>Catalysis of an enzymatic reaction
Tr	**Transcription regulation**<br>Physical binding of a transcription factor to target gene’s promoter
M	**MicroRNA binding**<br>Regulation of gene expression by binding of microRNA to target mRNA
**Functional interactions**	
IE	**Influence on expression**<br>Protein’s or compound’s action results in changing the expression level of target gene(s)
Cn	**Competition**<br>Protein activity regulation by competition at the substrate binding site
?	**Unspecified interactions**<br>Mechanism is unknown or/and effect is indirect
**Logical relations**	
GR	**Group relation**<br>Object belongs to a generic group of related objects
CS	**Complex subunit**<br>Protein is a subunit of a protein complex
SR	**Similarity relation**<br>Chemically similar compounds with chosen Tanimoto similarity score
